# The Critics of the Committee on Revision of the New Pharmacopœia

**Published:** 1883-03

**Authors:** 


### THE CRITICS OF THE COMMITTEE ON REVISION OF THE NEW PHARMACOPŒIA.

The committee charged with the arduous and responsible duty of revising and providing for the publication of the new pharmacopœia has reaped for its pains a large measure of severe abuse. The committee was composed, so far as we are informed, of upright and honorable men, and it has discharged the trust committed to its care in a praiseworthy and entirely commendable manner. In view of which fact, the untempered—not to say ill-tempered—criticisms which have been heaped upon it in certain quarters is not at all creditable to the source whence the attacks originated. These diatribes, sadly enough, partake of the vicious style of public denunciation and vilification so common in these degenerate days among hireling politicians and subservient political organs; they are altogether out of place in the colums of conservative scientific journals and reflect most injuriously upon the taste and judgment which permits their appearance there. Indeed the violence in which at least one of our respected cotemporaries has seen proper to indulge in the discussion of this subject, is calculated to excite the suspicion that its rage is the result rather of disapppointment at the direction given to the publication of the book than to a purely disinterested and laudable interest in the decision and action of the committee. Justice, wisdom and moderation would prompt a different and a better course, and we trust that these cardinal principles will ultimately prevail, not only in medical journalism, but in all things that pertain to the medical profession.

